# Oxidation of *Arabidopsis thaliana* COX19 Using the Combined Action of ERV1 and Glutathione

**DOI:** 10.3390/antiox12111949

**Published:** 2023-11-01

**Authors:** Flavien Zannini, Johannes M. Herrmann, Jérémy Couturier, Nicolas Rouhier

**Affiliations:** 1 Université de Lorraine, INRAE, IAM, F-54000 Nancy, France; flavien.zannini@univ-lorraine.fr (F.Z.); jeremy.couturier@univ-lorraine.fr (J.C.); 2 Cell Biology, University of Kaiserslautern, RPTU, 67663 Kaiserslautern, Germany; hannes.herrmann@biologie.uni-kl.de

**Keywords:** MIA40, ERV1, glutathione, oxidative folding, mitochondrial intermembrane space

## Abstract

Protein import and oxidative folding within the intermembrane space (IMS) of mitochondria relies on the MIA40–ERV1 couple. The MIA40 oxidoreductase usually performs substrate recognition and oxidation and is then regenerated by the FAD-dependent oxidase ERV1. In most eukaryotes, both proteins are essential; however, MIA40 is dispensable in *Arabidopsis thaliana*. Previous complementation experiments have studied yeast *mia40* mutants expressing a redox inactive, but import-competent versions of yeast Mia40 using *A. thaliana* ERV1 (AtERV1) suggest that AtERV1 catalyzes the oxidation of MIA40 substrates. We assessed the ability of both yeast and *Arabidopsis* MIA40 and ERV1 recombinant proteins to oxidize the apo-cytochrome reductase CCMH and the cytochrome *c* oxidase assembly protein COX19, a typical MIA40 substrate, in the presence or absence of glutathione, using in vitro cysteine alkylation and cytochrome *c* reduction assays. The presence of glutathione used at a physiological concentration and redox potential was sufficient to support the oxidation of COX19 by AtERV1, providing a likely explanation for why MIA40 is not essential for the import and oxidative folding of IMS-located proteins in *Arabidopsis*. The results point to fundamental biochemical differences between *Arabidopsis* and yeast ERV1 in catalyzing protein oxidation.

## 1. Introduction

Mitochondria are essential organelles, carrying out many crucial cellular pathways such as ATP production, apoptosis, iron–sulfur (Fe-S) cluster biogenesis, and ion homeostasis. Mitochondria are formed by two aqueous compartments, the matrix and the intermembrane space (IMS), separated by two biological membranes, the outer membrane (OM) and the inner membrane (IM). Among the ~1000 proteins present in this organelle, 51 and 127 proteins have been reported as present in the IMS of yeast and human mitochondria, respectively [1,2,3]. After their translation in the cytosol, the import of IMS-located proteins relies on at least three distinct pathways [4,5]. The predominant pathway allows both this import and oxidative folding [4]. Many proteins using this pathway are characterized by the presence of two repeated Cx_3_C (for TIM proteins) or Cx_9_C (for COX proteins) motifs [6,7], but other proteins (Atp23, CCS1, MICU, and SLP2) with particular cysteine motifs have proven to also be imported using this pathway [8,9,10,11,12].

The import and oxidative folding of these protein substrates is ensured by a pair of proteins referred to as the oxidoreductase Mitochondrial Intermembrane space import and Assembly protein 40 (MIA40) and the Essential for Respiration and Vegetative growth (ERV1) [13,14]. The former introduces disulfide bonds in protein substrates and is regenerated by the latter. The core structure of MIA40 is formed by two disulfide-bridged helices forming a hydrophobic cleft important for substrate recognition, and an N-terminal flexible arm containing the catalytic cysteines present in a CPC motif [7,15,16]. Three different models have been proposed to describe the molecular interactions between MIA40 and its substrates [17]. In the folding trap model, protein translocation into the IMS is dependent on the formation of disulfides on MIA40 substrates, preventing back-translocation into the cytosol. In the two so-called “trans-site receptor” models, substrate trapping is ensured initially by the hydrophobic domain of MIA40 but requires the oxidase activity of MIA40, either concomitantly or in a further separate step [7,17]. The hydrophobic cleft interacts with specific recognition sequences present in MIA40 substrates, referred to as MISS or ITS (for Mitochondrial IMS-Sorting Signal and Intermembrane space Targeting Signal, respectively) [18,19,20]. These specific motifs formed by hydrophobic and aromatic amino acids are situated in close proximity to the Cx_3_C or Cx_9_C motifs. After this initial step of recognition, the substrates are oxidized by the catalytic cysteine pair of MIA40, promoting their folding and retention in the IMS [7,21]. All of these proposed models agree in their idea that MIA40 is finally released in a reduced form after substrate oxidation. Its re-oxidation is achieved by the homodimeric FAD-dependent thiol oxidase, ERV1.

Most ERV1 isoforms possess two conserved cysteine pairs forming intramolecular disulfides. An exposed disulfide (shuttle disulfide) catalyzes the oxidation of the CPC disulfide of MIA40 [22,23,24,25] and is then regenerated by the disulfide present in the central domain of the second monomer. Electrons are then shuttled to the FAD cofactor, to cytochrome *c* and cytochrome *c* oxidase [26,27,28,29,30]. While the core domain of ERV1 proteins from different species is relatively well conserved, a noticeable difference exists among them. In opisthokonts, the catalytic disulfide is formed by cysteines present in a Cx_2_C motif found in the N-terminal part of ERV1, whereas it is formed between cysteines present in a Cx_3–5_C motif found in the C-terminal part in isoforms from plants and protists [31,32]. Proteins from protists have an additional KISS domain located between the FAD central domain and the catalytic domain [33]. In opisthokonts, both MIA40 and ERV1 have proven to be essential as knock-out mutants in yeast or in animal cells are lethal [16,34,35]. Only a few human patients bearing mutations affecting these proteins have been reported and they present severe diseases such as myopathy and neuronal cell degeneration [36]. The mutation of ERV1 is also lethal in *Arabidopsis thaliana* and in the protist *Leishmania infantum* [31,32]. However, several protists, including apicomplexan and kinetoplastid parasites, lack MIA40 [32,33]. Moreover, an *Arabidopsis* *mia40* mutant displays no growth phenotype, despite a slight decrease in complex I activity [37]. From a functional point of view, ERV1 proteins from these parasites could not functionally substitute the essential ERV1 in yeast [38] and *Trypanosoma brucei* ERV1 is unable to directly oxidize a small TIM protein in vitro [39]. This is different for *Arabidopsis* ERV1, since it partially complements the yeast *erv1* mutant [40]. It was shown that AtERV1 and ScMia40 interact together in vivo but that AtERV1 is unable to oxidize ScMia40 properly [40]. These incompatibilities have been explained by the structural divergence between ERV1 isoforms [31,40]. Accordingly, chimeric ERV1 proteins combining the N-terminal domain of yeast Erv1 and the core domain of *Leishmania tarentolae* ERV, thus mimicking the yeast Erv1 structural architecture, were able to complement the yeast *erv1* mutant [32]. The question of whether parasite and plant ERV1 proteins might exert the function of MIA40 was addressed by complementing *mia40* yeast cells. Whereas none of the tested truncated, chimeric or mutated ERV constructs from *L. tarentolae* were able to rescue these cells, AtERV1 could when a redox-inactive but chaperone-competent MIA40 protein was co-expressed [40]. Altogether, these observations suggested that AtERV1 was able to directly oxidize MIA40 substrates to some extent, as long as they are imported. Incidentally, this suggested that a non-redox-active receptor protein might be required in organisms lacking MIA40. Alternatively, a recent study identified Mic20, a thioredoxin-like protein belonging to the mitochondrial contact site and cristae organization system (MICOS) complex, as a potential candidate functionally replacing MIA40 in kinetoplastid parasites [41]. However, the protein was not identified in the interactome of *L. tarentolae* ERV and does not seem to be present in all protist lineages lacking MIA40 [42].

In order to further examine the biochemical properties of the plant ERV1-MIA40 system, we have compared the capacity of the *A. thaliana* and *S. cerevisiae* ERV1 to oxidize in vitro the *Arabidopsis* cytochrome *c* oxidase biogenesis factor COX19 in the presence or absence of the respective MIA40 proteins, but also CCMH, a reductase dedicated to the reduction of apo-cytochrome *c* in the IMS. Moreover, from the reports indicating that glutathione improves protein import both in vivo and in vitro by counteracting, in particular, the formation of long-lived or unproductive MIA40-substrate covalent intermediates [14,43], the possible contribution of glutathione was examined in these in vitro assays.

## 2. Materials and Methods

### 2.1. Cloning, Site-Directed Mutagenesis, Production and Purification of Recombinant Proteins

The coding sequence of *A. thaliana* COX19 (At1g69750) was amplified from leaf cDNAs using the following primers (restriction sites underlined): AtCOX19 for 5′ CCCCCCCCATATGAGTACAGGTGGAGCATTT 3′ and AtCOX19 rev 5′ CCCCGGATCCTCAATGTTCGATACTCTCTGT 3′. It was then cloned in the pET15b plasmid between NdeI and BamHI restriction sites, allowing the production of an N-terminal His-tagged protein. The protein was expressed in the *E. coli* Origami2 strain, cultivated at 37 °C in LB medium, and protein production was induced in exponential phases by adding 100 μM IPTG (isopropyl-β-D-thiogalactopyranoside) for 4 h before collecting by centrifugation bacterial cells, which were resuspended in a 50 mM Tris-HCl pH 8.0, 300 mM NaCl, 10 mM imidazole buffer. Cell lysis and IMAC protein purification of His-tagged COX19 was performed using a procedure similar to the one previously described for other proteins [44]. The mutagenesis of both cysteines present in the CERC and CEQKSC active sites of AtERV1 into serines was carried out by primer extension using the following two complementary mutagenic primers: AtERV1 SERS for 5′ TCCCGAATGTATCCTTCTAGAGAATCTGCGGATCACTTCAAA 3′, AtERV1 SERS rev 5′ TTTGAAGTGATCCGCAGATTCTCTAGAAGGATACATTCGGGA 3′, AtERV1 SEQKSS for 5′ TGGGGCAAGTTAGAGTCTGAGCAGAAAAGTTCTGATCTCCATGGAACT 3′, and AtERV1 SEQKSS rev 5′ AGTTCCATGGAGATCAGAACTTTTCTGCTCAGACTCTAACTTGCCCCA 3′. In the first round of PCR, two fragments with overlapping ends were generated by using forward and mutagenic reverse primers and reverse and mutagenic forward primers, respectively. For the second round of PCR, these fragments were mixed for 10 PCR cycles without primers, before adding ERV1 forward and reverse primers for 25 additional PCR cycles to obtain the final product with the desired mutation. Finally, PCR fragments were cloned in the pET12a plasmid between NdeI and BamHI restriction sites. The corresponding variants were named AtERV1 SEQKSS and AtERV1 SERS. These two variants, as well as recombinant *S. cerevisiae* and *A. thaliana* ERV1 and MIA40 and *A. thaliana* CCMH, were expressed in the *E. coli* BL21(DE3) strain from pET24a-ScErv1, pGEX6-ScMia40, pET12a-AtMIA40, pET12a-AtERV1, pET12a-AtERV1 SEQKSS, pET12a-AtERV1 SERS, and pQE60-AtCCMH plasmids, following procedures described previously [7,16,29,40,43,45]. Protein concentrations were determined spectrophotometrically using the respective molar extinction coefficients at 280 nm, calculated from the amino acid sequences using the Protparam tool (AtMIA40, 7365 M^−1^·cm^−1^; AtERV1 and its variants, 50,063 M^−1^·cm^−1^; ScMia40, 11,835 M^−1^·cm^−1^; ScErv1, 42,315 M^−1^·cm^−1^; AtCCMH, 3105 M^−1^·cm^−1^; AtCOX19, 3230 M^−1^·cm^−1^).

### 2.2. Reduction of COX19 and CCMH Proteins

Before each test, around 1 mg of COX19 and CCMH proteins was reduced using a 50-fold excess dithiothreitol (DTT) for 4 h at room temperature, then desalted against 50 mM phosphate buffer pH 7.4 using a Sephadex-G25 column (Sigma-Aldrich, Burlington, MA, USA).

### 2.3. Reduction of Cytochrome c

The reduction of 20 µM cytochrome *c* from equine heart (SIGMA-Aldrich) was followed spectrophotometrically by recording the absorbance changes at 550 nm over time, usually 60 min (Cary 50 Variant-Agilent, Santa Clara, CA, USA). The reaction was performed in 500 µL of 50 mM phosphate buffer at pH 7.4, in the presence of 40 µM reduced *Arabidopsis* COX19 or CCMH and various combinations of *A. thaliana* and *S. cerevisiae* ERV1 and MIA40 at 4 µM. A control was performed in the presence of 40 µM DTT. All experiments were repeated at least three times and the data shown are representative of the results obtained.

### 2.4. Alkylation Shift Experiments for Redox State Detection

For all tests, 40 µM reduced proteins were incubated with 4 µM ERV1, alone or in presence of 4 µM MIA40, for different amounts of time in 50 µL of 50 mM phosphate buffer pH 7.4. After a precipitation with 10% TCA, the reduced cysteines were alkylated with methyl-polyethylene glycol-maleimide of 1.2 kDa (mmPEG_24_) or 2 kDa (mPEG_2000_) and 2.5 µg of proteins loaded on non-reducing SDS-PAGE 17%, as described previously [46]. The oxidation of AtCOX19 was also assessed in the presence of an 8 mM GSH/GSSG mixture corresponding to a redox potential of −274 mV at pH 7.4. In fact, this solution corresponded to a freshly prepared GSH solution in which we had estimated that 0.25% GSSG was present. This estimate was determined from a spectrophotometric detection in an assay containing 200 µM NADPH and 0.5 unit of baker yeast glutathione reductase (GR, Sigma-Aldrich).

### 2.5. Redox Potential Measurements

The midpoint redox potentials of AtMIA40 and both AtERV1 SEQKSS and SERS variants were determined by oxidation–reduction titrations using monobromobimane (mBBr) labeling, after incubation of the oxidized proteins for 2 h in mixtures of 2 mM oxidized and reduced DTT at defined redox potentials, as described previously [43]. Because of a rapid FAD-mediated re-oxidation of the CERC disulfide present in the SEQKSS variant, its titration was performed under anaerobiosis in a glove box with less than 3 ppm O_2_, whereas the other titrations were carried out aerobically.

## 3. Results and Discussion

### 3.1. ERV1 Is Not Able to Directly Oxidize COX19 In Vitro

In order to assess the capacity of yeast and *A. thaliana* ERV1 proteins alone or in the presence of MIA40 to oxidize reduced AtCOX19, all proteins were expressed as recombinant proteins in *E. coli*. The yeast and *Arabidopsis* MIA40 and ERV1 proteins were expressed in the regular *E. coli* BL21(DE3) strain, whereas AtCOX19 was expressed in the more oxidizing cytoplasmic compartment of the *E. coli* Origami2 strain. Nonetheless, all proteins were purified in their oxidized forms. Since AtCOX19 has proven to be resistant to reduction and to re-oxidize quite quickly, its reduction was achieved with a 50-fold excess of DTT for 4 h. It is worth noting that AtCOX19 possesses four cysteinyl residues forming two intramolecular disulfides. Two types of in vitro assays have been carried out. The first method measured AtCOX19 oxidation in a coupled assay by measuring cytochrome *c* reduction at 550 nm over time with catalytic amounts of ERV1 and/or MIA40. Using similar reaction mixtures, the second method assessed the AtCOX19 redox state after cysteine alkylation and separation on non-reducing SDS-PAGE. In this assay, only the reduced cysteine residues were accessible for modification. This resulted in a change in the migration profile that allows distinguishing different redox forms, i.e., completely reduced or with one or two intramolecular disulfides. For comparison, both assays were performed in the same time frame.

**Figure 1:**
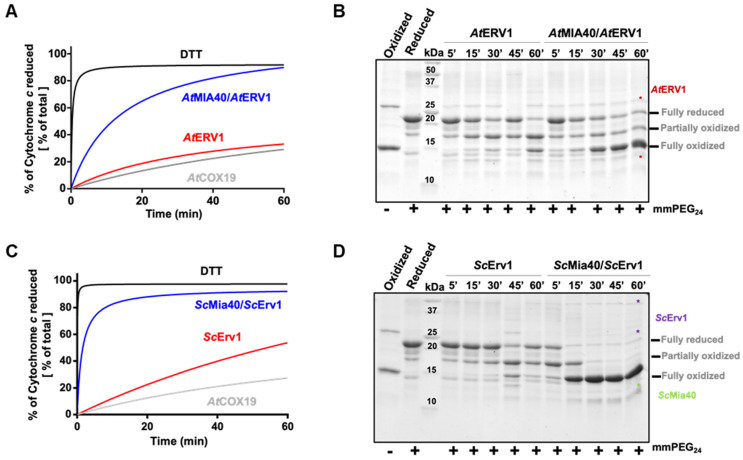
In vitro oxidation of *Arabidopsis* COX19 protein using the ERV1–MIA40 couple from *Arabidopsis thaliana* or *Saccharomyces cerevisiae*. Capacity of ERV1 alone or in the presence of MIA40 to oxidize *Arabidopsis* COX19 using *A. thaliana* (**A**,**B**) or *S. cerevisiae* (**C**,**D**) proteins. In (**A**,**C**), the electron transfer from reduced COX19 (40 µM, grey line) was followed by recording the reduction of 20 µM cytochrome *c* at 550 nm over time in the presence of 4 µM ERV1 alone (red line) or with 4 µM MIA40 (blue line). A reference was made using 40 µM DTT as an electron donor (black line). In (**B**,**D**), COX19 (40 µM) oxidation was followed over time using cysteine alkylation using mmPEG_24_ and SDS-PAGE separation after incubation with 4 µM ERV1 alone or in the presence of 4 µM MIA40.

In the cytochrome *c* reduction assay, it became apparent that AtERV1 has no or a very poor capacity to oxidize AtCOX19 (Figure 1A, Figure S1), the presence of AtMIA40 being critical to reach a good oxidation efficiency, as compared with the control test performed using DTT instead of AtCOX19 and both AtERV1 and AtMIA40 proteins. The same conclusion arose from the alkylation shift assay (Figure 1B). Indeed, in the presence of AtERV1 alone, there were only minimal amounts of completely oxidized AtCOX19 for up to 45 min; instead, there was formation of a partially oxidized intermediate with one disulfide. Only after 1 h were higher amounts of completely oxidized AtCOX19 accumulated. On the contrary, the combined presence of AtMIA40 and AtERV1 promoted a near-complete AtCOX19 oxidation at the end of the 1 h reaction. Globally, similar results have been obtained when yeast Mia40 and Erv1 proteins have been used (Figure 1C,D). ScErv1 alone cannot efficiently oxidize AtCOX19 in the absence of ScMia40. In both assays, the reactions were even more efficient, with a complete AtCOX19 oxidation after 30 min. This difference in efficiency between the plant and yeast oxidation systems might be due to an intrinsic difference between both ERV1 proteins, as has been observed previously when their activity was tested in the cytochrome *c* reduction assay performed in the absence of MIA40 [40]. In addition, these results pointed to an indispensable contribution of MIA40 for COX19 oxidation in these in vitro conditions.

### 3.2. Arabidopsis ERV1 Oxidizes COX19 in the Presence of GSH and MIA40 Becomes Dispensable

While the in vitro results shown in Figure 1 point to the requirement of a redox-active AtMIA40 for AtCOX19 oxidation by AtERV1, previous observations indicated that AtERV1 was able to complement a yeast *mia40* mutant expressing a redox-inactive Mia40 and suggested the existence of an oxidase activity of AtERV1. We hypothesize that a redox component could be missing in our in vitro assays when compared with the yeast cellular context. It was shown that a glutathione pool is present in the IMS, equilibrating with the cytosolic glutathione pool due to the passive exchange of GSH via porins [47]. In fact, rather elevated glutathione concentrations (5 to 13 mM) exist in the IMS, with measured redox potentials comprised between −255 and −300 mM in yeast cells and of *ca* −290 mV in human cells [14,47,48,49]. Also, the GSH pool influences the MIA40 redox state, although MIA40 is only slowly reduced by GSH [43,47]. With regard to the report stating that low amounts of Grx2 are present in the mitochondrial IMS of yeast, it was proposed that Grx2 mediates this GSH effect [50,51]. Hence, the presence of these components may ensure some sort of proofreading activity for misfolded proteins or avoiding protein misfolding at the import step [43], in a way somehow similar to the oxidative protein folding occurring in the ER, a process in which protein disulfide isomerase (PDI) activity is assisted by GSH [52]. Nevertheless, contrasting observations exist. While some in vivo and in vitro studies have indeed shown positive effects of GSH [14,43], other studies have reached the opposite conclusion: that GSH inhibits the import and/or oxidation of some MIA40 substrates [53]. This might indicate that changes in the concentrations and redox state of glutathione are likely critical factors for in vivo, but also for in vitro, assays.

**Figure 2:**
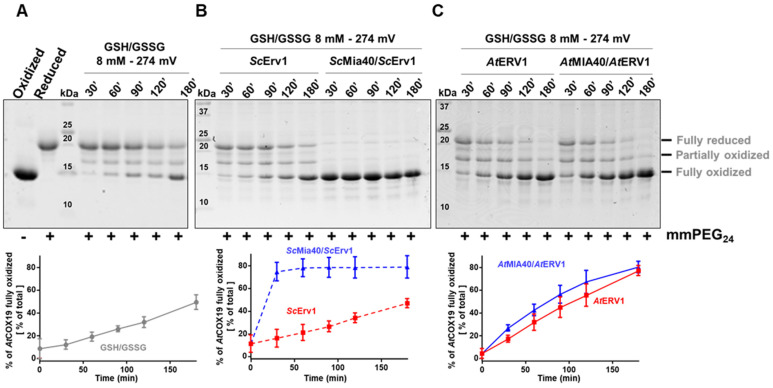
In vitro oxidation of *Arabidopsis* COX19 protein using the ERV1–MIA40 couple from *Arabidopsis thaliana* or *Saccharomyces cerevisiae* in the presence of glutathione. The oxidation of AtCOX19 (40 µM) was followed over time using a cysteine alkylation assay (mmPEG_24_ alkylation and SDS-PAGE separation) in the presence of a total glutathione concentration of 8 mM, adjusted to a midpoint redox potential for the GSH/GSSG couple of—274 mV at pH 7.4 (**A**). The capacity of *Arabidopsis* or of yeast ERV1 alone, or in presence of MIA40, each at a 4 µM concentration, was tested in the same conditions (**B**,**C**). The amount of fully oxidized AtCOX19 relative to the total AtCOX19 amounts (represented by a sum of the intensity of the three bands corresponding to the various redox states of AtCOX19) was quantified in each condition. Mean values ± SD of at least three replicates are shown.

The impact of glutathione on AtCOX19 oxidation was thus examined over a longer period, typically 3 h, to reach equilibrium, using a concentration of 8 mM and a GSH/GSSG ratio corresponding to a redox potential of −274 mV at pH 7.4. The timeframe and redox potential of the glutathione were chosen based on previous studies (see above) and were somehow imposed by the presence of 0.25% GSSG in freshly prepared GSH solutions. At these values, a significant oxidation of AtCOX19 occurred when only glutathione was present, with the fully oxidized form representing *ca* 40% of the total AtCOX19 at the end of this experiment (Figure 2A). Hence, the low amounts of GSSG and/or the combination of GSH and O_2_ was, to some extent, sufficient to introduce both disulfides in AtCOX19. Then, the impact of this GSH/GSSG buffering solution was assayed in the presence of the yeast (Figure 2B) and *Arabidopsis* (Figure 2C) MIA40–ERV1 couples. ScErv1 was unable to oxidize AtCOX19 in the presence of glutathione and the reaction still required ScMia40 (around 90% of AtCOX19 oxidation after 30 min, as observed in Figure 1). On the contrary, AtERV1 was able to oxidize AtCOX19 in the presence of glutathione (80% of oxidized AtCOX19 after 3 h) and the reaction was not improved by the presence of AtMIA40 (Figure 2C). Overall, unlike yeast proteins, there is a clear effect of GSH on *Arabidopsis* MIA40–ERV1 oxidizing properties, with GSH or GSSG supporting ERV1 oxidase activity and maybe counteracting MIA40 activity. In this respect, it would also be informative to measure the redox potential of the MIA40 catalytic disulfide. By comparing it with the one of glutathione in the IMS of plant mitochondria, it may help to determine to which extent MIA40 is oxidized in plant cells. A recent study concluded that the in vivo redox state of Mia40 at steady-state in yeast cells is about 70% oxidized and that it is influenced both by Erv1 and glutathione [47].

### 3.3. Arabidopsis MIA40 Oxidase Activity Is Modulated upon Interaction with ScErv1

**Figure 3:**
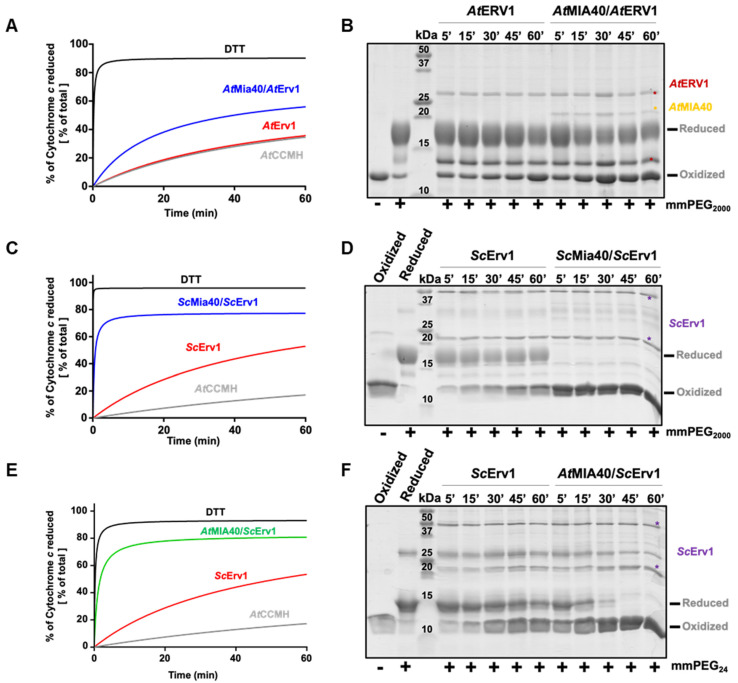
In vitro oxidation of *Arabidopsis* CCMH using the ERV1–MIA40 couple from *Arabidopsis thaliana* or *Saccharomyces cerevisiae*. Capacity of *A. thaliana* (**A**,**B**) or *S. cerevisiae* (**C**,**D**) ERV1–MIA40 couples to oxidize *Arabidopsis* CCMH using cysteine alkylation with mPEG2000 or mmPEG24 (as indicated) and SDS-PAGE separation or cytochrome *c* reduction. In all of these assays, 40 µM CCMH was incubated with 4 µM ERV1 alone or in presence of 4 µM MIA40. In (**E**,**F**), the same tests were performed using a hybrid system comprising *S. cerevisiae* Erv1 and *A. thaliana* MIA40.

Unlike those in yeast and humans, the maturation of *c* type cytochromes in the IMS of plant mitochondria requires a reducing component called CCMH [54]. This protein possesses a pair of catalytic cysteines, present in a Cx_2_CH motif, that allows apo-cytochrome *c* reduction before heme incorporation [45]. Since we had observed that the yeast Mia40–Erv1 couple was more efficient in oxidizing AtCOX19, which suggested that these proteins have different redox properties, we analyzed whether both the yeast and *Arabidopsis* MIA40–ERV1 couples can oxidize CCMH. Using the same read-outs (cytochrome *c* reduction and cysteine alkylation assays) (Figure 3A,B, Figure S2), we observed that AtERV1 was unable to oxidize AtCCMH and that adding AtMIA40 resulted in very inefficient reactions, as most AtCCMH remained in a reduced state after 1 h. On the contrary, a complete oxidation of AtCCMH was visible in less than 5 min in both assays when using the yeast counterparts (Figure 3C,D). This oxidation required the presence of both proteins, as ScErv1 alone did not have the capacity to efficiently oxidize AtCCMH. These data indicate that one of the yeast oxidases possesses particular redox properties that the plant orthologs do not have, and prompted us to perform a test in which AtMIA40 was combined with ScErv1. The other combination cannot be tested as we previously observed that AtERV1 does not have the capacity to properly oxidize ScMia40 [40]. With this combination, AtMIA40 was now able to oxidize AtCCMH efficiently (Figure 3E,F), although the reaction appeared slightly less efficient than with the complete yeast system. This unexpected observation should not (or not only) correspond to a better efficiency of ScErv1 to oxidize AtMIA40, but may indicate that ScErv1 participates in the oxidation reaction by acting on AtCCMH, AtMIA40, or both, thus promoting MIA40 activity. It has been proposed in some studies that ternary complexes are formed in yeast involving Erv1, Mia40, and the substrate [55,56].

### 3.4. Redox Potentials of the Disulfide Bridges Present in Arabidopsis ERV1/MIA40 Proteins

**Figure 4:**
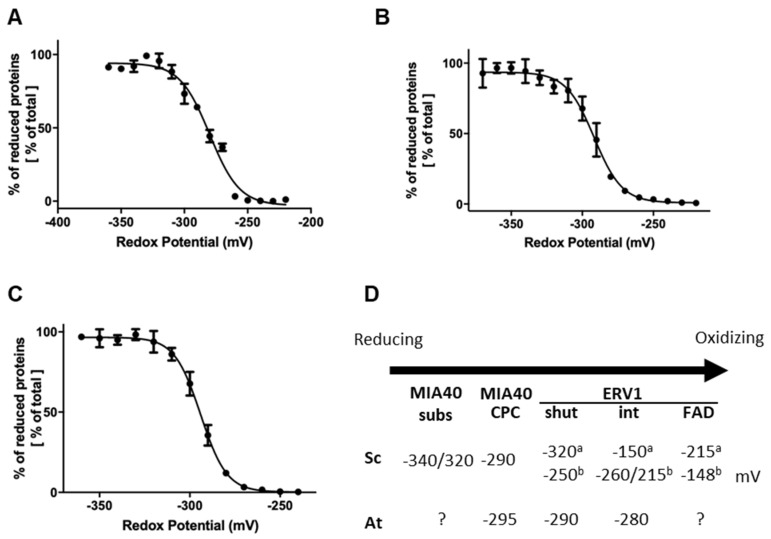
Redox titration of the catalytic disulfides in AtERV1 and AtMIA40. The titrations of the dithiol-disulfide couples of AtERV1 SEQKSS (**A**), AtERV1 SERS (**B**), and AtMIA40 (**C**) were carried out using mixtures of reduced and oxidized DTT for a total DTT concentration of 2 mM for 2 h at pH 7.0. Free thiol groups were labeled using mBBr and the resulting fluorescence emission was expressed as % of maximal fluorescence and fitted to the redox potential of the solution. The obtained E_m_ value is the mean ± SD of three replicates. (**D**) Summary of the midpoint redox potentials measured for the disulfides of MIA40 substrates, MIA40 CPC active motif, and ERV1 from *S. cerevisiae* (Sc) and *A. thaliana* (At). For ERV1, we have distinguished the shuttle (shut) and internal (int) disulfides and added the redox potential of the FAD/FADH_2_ couple when known. Different values, labeled a or b, have been obtained for ScERV1 and are based, respectively, on references [57] or [58].

We have previously observed in a cytochrome *c* reduction assay that AtERV1 oxidizes reduced glutathione, or, in other words, that AtERV1 is reduced by GSH, whereas ScErv1 has a poor capacity to do so [40], which is actually confirmed by the results presented in Figure 2. The absence of an additive effect of MIA40 in the ERV1- and GSH-mediated oxidation of AtCOX19 using *Arabidopsis* proteins, when compared with the yeast proteins, also suggests that the MIA40 redox properties differ between both organisms. In fact, GSH does not reduce the yeast Mia40 protein very well [43]. While MIA40 from both species have a rather comparable structural organization, ERV1 proteins differ, as already mentioned, in the positions of their so-called shuttle disulfide (N-terminal region for ScErv1 vs. C-terminal region for AtERV1) and cysteine spacing residues (two residues for ScErv1 vs. four residues for AtERV1), whereas the signature and position of the internal disulfide are unchanged. Of note, the resolution of the 3D structure of AtERV1 indicates that the shuttle disulfide is situated in a flexible or unstructured region [30]. Hence, the difference in redox properties between both proteins is likely due to the position and/or the redox potential of the shuttle disulfide. We measured the redox potentials of the disulfides in AtMIA40 and AtERV1. For the latter, we generated variants of AtERV1 retaining one of the cysteine pairs. The AtERV1 SEQKSS variant, in which both external cysteines are substituted by serines, retains the internal disulfide, whereas the AtERV1 SERS variant retains the shuttle disulfide. Very similar values were obtained for all proteins at pH 7, i.e., −295 mV for AtMIA40, −290 mV for the shuttle disulfide of AtERV1, and −280 mV for the internal disulfide of AtERV1 (Figure 4). Therefore, when only considering the thermodynamic aspects, these values fit with the disulfide-relay reactions during which redox potential decreases from the substrate to the CPC motif of MIA40 and then to ERV1 disulfides. As a case for comparison, the midpoint redox potential of the catalytic disulfide of ScMia40 was determined at −290 mV at pH 7.0 [29]. Concerning ScErv1, contrasting results have been obtained in two different studies. In the first study, the thermodynamic of the electron transfer reactions was not linear since the redox potential of the shuttle disulfide of ScErv1 was −320 mV, whereas that of the internal disulfide close to the FAD moiety was −150 mV, and that of the FAD itself was −215 mV [57]. In the subsequent study, in which measurements were achieved under anaerobic conditions, the obtained values were thermodynamically favorable, with values of −250 mV for the shuttle disulfide, between −260 mV and −215 mV for the internal disulfide, and −148 mV for FAD [58].

### 3.5. Model for the Oxidation of COX19 with AtERV1 in the Presence of GSH

**Figure 5:**
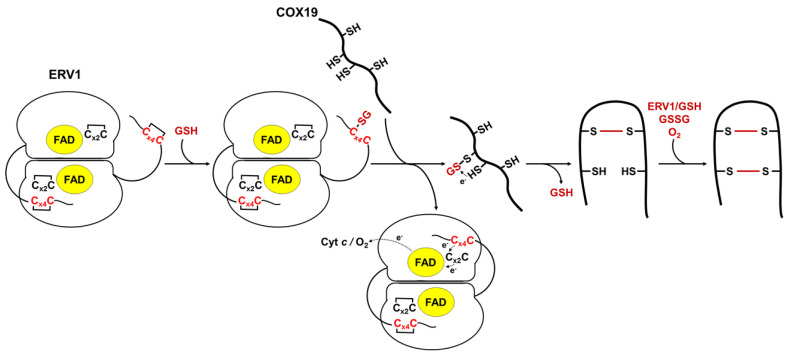
Proposed model of GSH- and AtERV1-mediated oxidation of *Arabidopsis* COX19 in the absence of MIA40. In this scheme, reduced glutathione (GSH) reacts with the shuttle disulfide of ERV1 (Cx_4_C) (rather than the catalytic disulfide, which is not in contact with the substrates), leading to the formation of a glutathione adduct on one of these cysteines. The glutathione molecule would then be transferred to one of the cysteine residues, forming the more internal disulfide in the substrates, to promote disulfide bond formation and the release of GSH. The second disulfide bridge of the substrate could be formed using the same mechanism (ERV1 + GSH) or, eventually, by the action of GSSG or of O_2_ in conjunction with metal traces. The shuttle disulfide of ERV1 is subsequently regenerated by transferring electrons to the catalytic Cx_2_C motif, FAD and oxygen (O_2_) or cytochrome *c* (cyt *c*).

We have built a hypothetical model proposing that an oxidized AtERV1 mediates AtCOX19 glutathionylation in the presence of reduced glutathione, without the need of AtMIA40 (Figure 5). The recognition of MIA40 substrates by ERV1 makes sense based on the very similar 3D structures adopted by MIA40 and its substrates. In fact, despite the fact that the redox potentials of the shuttle disulfide of AtERV1 and ScErv1 are in the same range, the effect of GSH on the capacity of both proteins to oxidize AtCOX19 is different.

In this model, GSH would react with the exposed shuttle disulfide of AtERV1, generating a glutathionylated ERV1 intermediate. We do not exclude the possible contribution of the catalytic disulfide; this might react first with GSH, but then the glutathione would have to be transferred to the shuttle disulfide anyway. Then, the glutathionylated ERV1 intermediate would react with a thiol group of the protein substrate, leading first to the substrate glutathionylation and then to the formation of an intramolecular disulfide, thus releasing GSH but not disulfide glutathione. In this context, this should not change the GSH/GSSG ratio and thus should not affect the glutathione redox potential in the IMS. Complete substrate oxidation could then proceed via oxygen, as proposed previously [15], or via another turnover involving GSH and AtERV1, or even oxidized glutathione. Note that in the assay without cytochrome *c*, AtERV1 should pass electrons to molecular oxygen to become re-oxidized. An alternative mechanism may be considered in which GSH participates in the reduction of disulfides formed between ERV1 and MIA40 substrates.

## 4. Conclusions

The results presented in this study highlight the specificities of the plant ERV1–MIA40 pathway towards GSH and two IMS proteins, COX19 and CCMH. It is first interesting to note that the *Arabidopsis* ERV1–MIA40 couple is not able to oxidize CCMH well. This may explain why no reducing system has been identified in plants so far, unlike in bacteria such as *E. coli*, where reduction of the periplasmic ortholog is assisted by CcmG using electrons coming from the cytoplasm and transferred via the membrane CcdA protein [59]. One of the differences between these systems is the presence of high GSH concentrations in the IMS and, eventually, of glutaredoxins, although this remains to be established for plants. The next step may be to verify the capacity of GSH in vitro, alone or with glutaredoxin, to reduce CCMH. The second important conclusion is the observation that the in vitro requirement of MIA40 for COX19 oxidation is abolished when ERV1 and physiological concentrations of glutathione provided at a relevant redox potential are present. The capacity of AtERV1 to bypass the AtMIA40 function in vitro may actually explain why AtERV1 was able to complement a yeast *mia40* mutant, restoring the amount of MIA40 substrates [40], and why the role of AtMIA40 is dispensable in *A. thaliana*, but not in opisthokonts.

## Data Availability

All data are presented in the manuscript. The data presented in this study are available on request from the corresponding author.

## Supplementary Materials

The supporting information can be downloaded at: https://www.mdpi.com/article/10.3390/antiox12111949/s1, Figure S1: In vitro oxidation of Arabidopsis COX19 protein by the ERV1-MIA40 couple from *Arabidopsis thaliana* or *Saccharomyces cerevisiae*; Figure S2: In vitro oxidation of *Arabidopsis* CCMH by the ERV1-MIA40 couple from *Arabidopsis thaliana* or *Saccharomyces cerevisiae*.
