# Supplementary material for: Oxidation of Arabidopsis thaliana COX19 Using the Combined Action of ERV1 and Glutathione
Source: Antioxidants (Basel). 2023 Nov 1;12(11):1949. doi: 10.3390/antiox12111949 (PMC10669224; doi:10.3390/antiox12111949)
Supplement: Supplementary file 1 [file antioxidants-12-01949-s001.zip › antioxidants-2675942-supplementary.pdf]

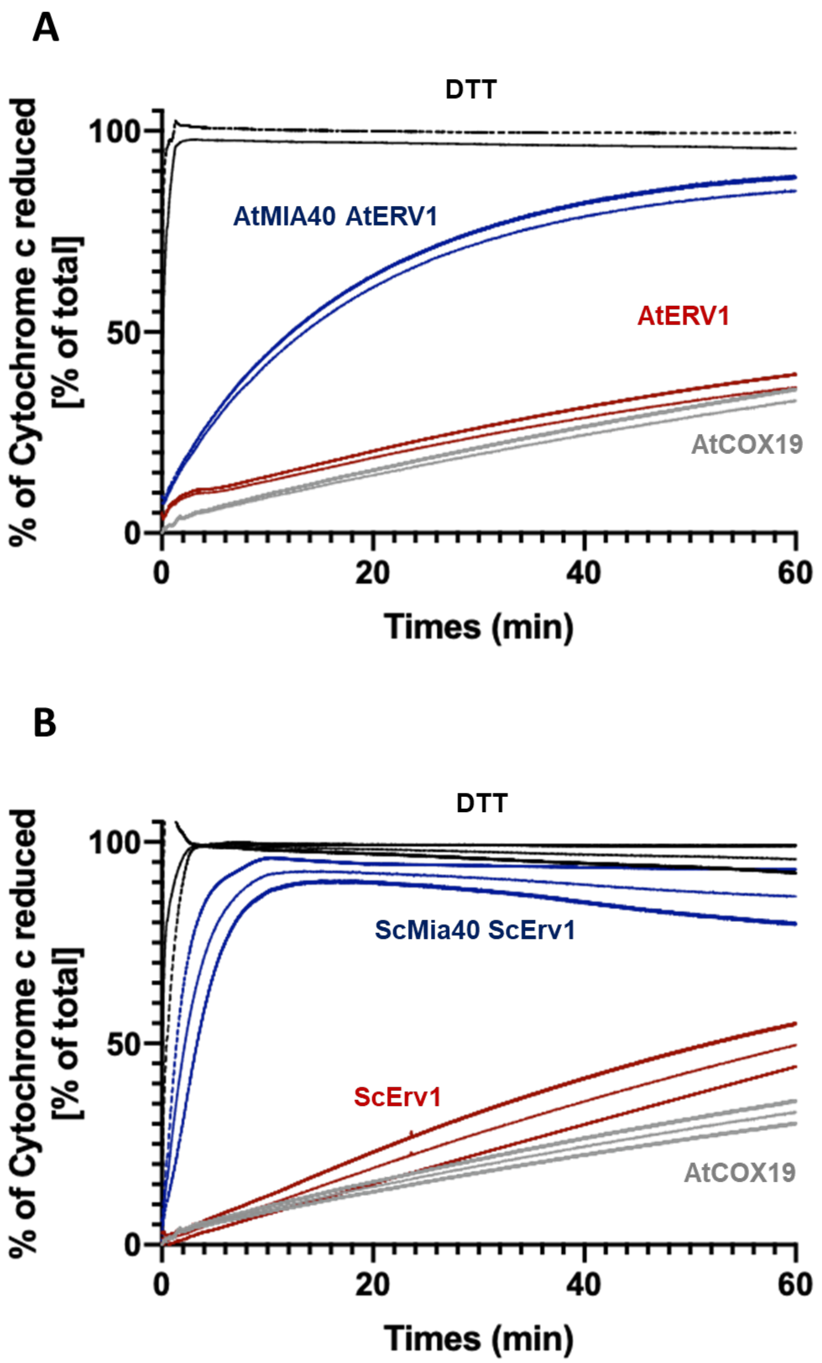

Supplementary Figure S1. *In vitro* oxidation of Arabidopsis COX19 protein by the ERV1-MIA40 couple from *Arabidopsis thaliana* or *Saccharomyces cerevisiae*.
Capacity of ERV1 alone or in the presence of MIA40 to oxidize Arabidopsis COX19 using *A. thaliana* (A) or *S. cerevisiae* (B) proteins. The electron transfer was followed by recording the reduction of 20 µM cytochrome c at 550 nm over time using different mixtures of reduced COX19 (40 µM), ERV1 (4 µM) and MIA40 (4 µM). References were made using 40 µM DTT as an electron donor (black lines) and they are similar as those shown in Figure S2A and B. Three repetitions are shown here in support of results shown in Figure 1.

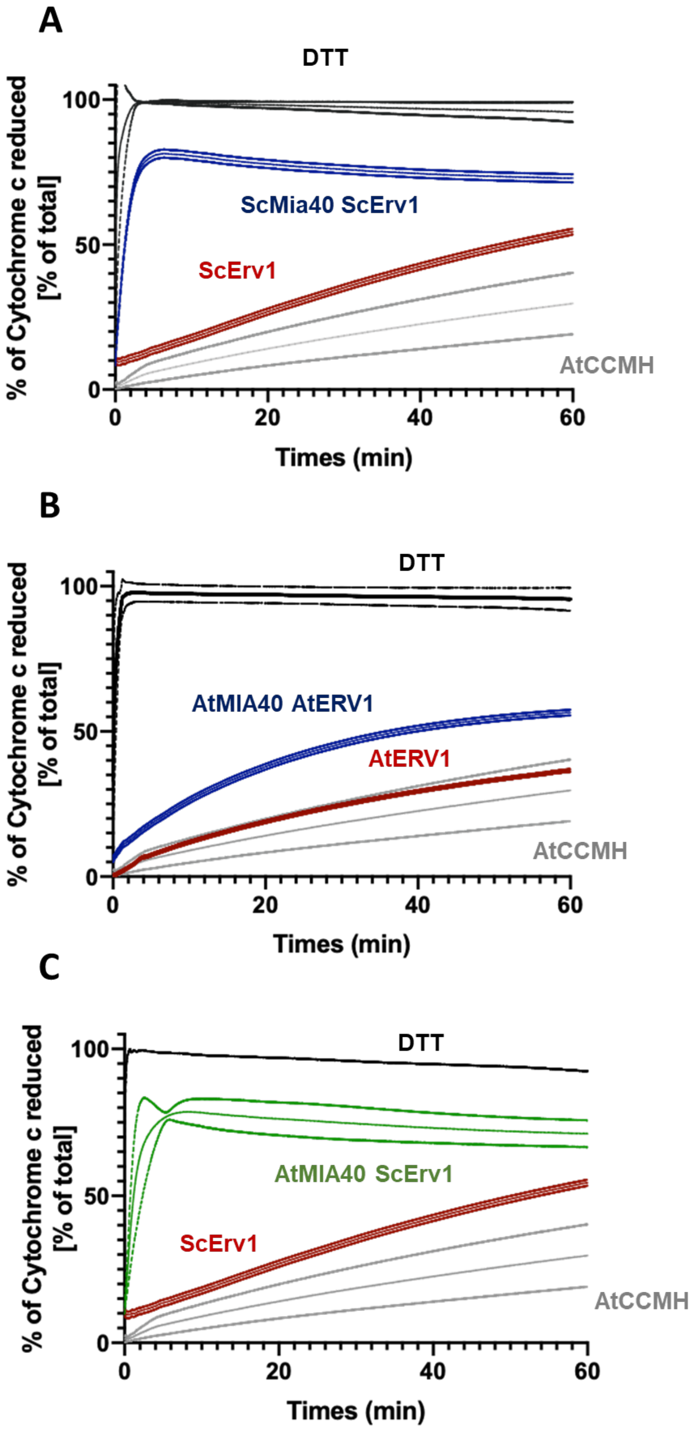

Supplementary Figure S2. In vitro oxidation of *Arabidopsis* CCMH by the ERV1-MIA40 couple from *Arabidopsis thaliana* or *Saccharomyces cerevisiae*.

Capacity of *A. thaliana* (A) or *S. cerevisiae* (B) ERV1/MIA40 couples to oxidize Arabidopsis CCMH using cytochrome c reduction assay. In C, the same tests were performed using a hybrid system comprising *S. cerevisiae* ERV1 and *A. thaliana* MIA40. In all these assays, the concentrations used are 40 µM for CCMH, and 4 µM for ERV1 and MIA40. References were made using 40 µM DTT as an electron donor (black lines). Three repetitions are shown here in support of results shown in Figure 3. The controls for AtCCMH have been repeated in all panels and the ones for ScERV1 in panels A and C.
